# Disrupted Resting Frontal–Parietal Attention Network Topology Is Associated With a Clinical Measure in Children With Attention-Deficit/Hyperactivity Disorder

**DOI:** 10.3389/fpsyt.2019.00300

**Published:** 2019-05-10

**Authors:** Yanpei Wang, Fuxiang Tao, Chenyi Zuo, Maihefulaiti Kanji, Mingming Hu, Daoyang Wang

**Affiliations:** ^1^State Key Laboratory of Cognitive Neuroscience and Learning, Beijing Normal University, Beijing, China; ^2^IDG/McGovern Institute for Brain Research, Beijing Normal University, Beijing, China; ^3^College of Educational Science, Anhui Normal University, Wuhu, China; ^4^The Key Laboratory of Mental Development and Learning Science, Xinjiang Normal University, Urumqi, China

**Keywords:** attention-deficit/hyperactivity disorder, frontal–parietal attention network, small world, minimum spanning tree, resting connectivity

## Abstract

**Purpose:** Although alterations in resting-state functional connectivity between brain regions have been reported in children with attention-deficit/hyperactivity disorder (ADHD), the spatial organization of these changes remains largely unknown. Here, we studied frontal–parietal attention network topology in children with ADHD, and related topology to a clinical measure of disease progression.

**Methods:** Resting-state fMRI scans were obtained from New York University Child Study Center, including 119 children with ADHD (male n = 89; female n = 30) and 69 typically developing controls (male n = 33; female n = 36). We characterized frontal–parietal functional networks using standard graph analysis (clustering coefficient and shortest path length) and the construction of a minimum spanning tree, a novel approach that allows a unique and unbiased characterization of brain networks.

**Results:** Clustering coefficient and path length in the frontal–parietal attention network were similar in children with ADHD and typically developing controls; however, diameter was greater and leaf number, tree hierarchy, and kappa were lower in children with ADHD, and were significantly correlated with ADHD symptom score. There were significant alterations in nodal eccentricity in children with ADHD, involving prefrontal and occipital cortex regions, which are compatible with the results of previous ADHD studies.

**Conclusions:** Our results indicate the tendency to deviate from a more centralized organization (star-like topology) towards a more decentralized organization (line-like topology) in the frontal–parietal attention network of children with ADHD. This represents a more random network that is associated with impaired global efficiency and network decentralization. These changes appear to reflect clinically relevant phenomena and hold promise as markers of disease progression.

## Introduction

Even when in the resting state and not performing any task processing, the brain is still working. The spontaneous brain activity present in the resting state is not random and usually shows high temporal coupling across different brain regions. This creates a set of brain networks that are densely interconnected and distinct from others. These resting-state networks are not a reaction to any task, but are intrinsically generated by the brain itself (1, 2). To date, at least seven brain networks have been identified by a variety of functional connectivity analysis approaches, and they show a high stability across individuals (1–6). The frontal–parietal attention network (FPAN) is involved cognitive process, especially attention in these networks (7–9). The FPAN not only has been studied in task-related activation studies involving sustained attention, but has also been confirmed by functional connectivity at rest that directly relates to attention performance (8, 10).

Graph theory has been used to study the architecture of brain networks (11, 12) and has revealed an economical and highly efficient organization of functional connectivity that combines global efficiency and local integration. This is called small-world (SW) topology and is characterized by limited long-distance and dense local connections (13, 14). Many brain diseases have been related to disrupted organization of brain networks. The study of brain networks has increased our understanding of the underlying pathophysiological mechanisms for many brain diseases such as epilepsy and schizophrenia (15–17). Attention-deficit/hyperactivity disorder (ADHD) is one of the most common psychiatric disorders during childhood and persists into adolescence and adulthood (18). Several whole-brain studies using graph theory analysis have reported a shift from a SW topology towards a more regular organization in ADHD, which results in increased local integration and loss of global network efficiency (19, 20). In addition, a vulnerability of some hub regions has been reported (21). As a neurodevelopmental disorder, ADHD is characterized by developmentally inappropriate symptoms of excessive inattention, impulsivity, and hyperactivity. Many studies have found that ADHD is a developmental disorder and associated with developmental delay (22). Recently, graph analysis studies have confirmed a shift from more random to more regular SW topological structure during maturation (23–25). Smit and colleagues have confirmed connectivity alteration that reflected increased network randomness, or decreased order (26). These results suggest that the maturational delay in ADHD is reflected by more random brain connectivity, but not more regular (23–25).

It is difficult to compare networks reported in graph theory studies across different groups and conditions. A normalization step is required to allow comparison. Common approaches are thresholding and/or comparing the observed network with randomized networks generated from the observed network; however, these do not provide a unique or consistent solution (27). One potential solution is minimum spanning tree (MST), which is derived from a weighted network (28). MST is an acyclic subnetwork that connects the same number of nodes and connections, and therefore not only makes the comparison of network topology easier across conditions but also avoids potential deviations that may be introduced through normalization steps. Several studies have used the MST approach to investigate brain networks and have shown that this approach is sensitive to brain disease, such as Alzheimer’s disease (29), epilepsy (30), and maturation from childhood into adulthood (23).

The aim of the present study was to explore the alteration of the FPAN connectivity or topology in children with ADHD. Increasing evidence has demonstrated that ADHD was a developmental disorder and associated with developmental delay. Typical maturation during childhood involves a shift from a random towards more regular networks (31). We hypothesized that, in youth with ADHD, functional networks would shift towards being more random, evidenced by decreased local integration and global efficiency. Although some previous studies report a regular topology in ADHD, with increased local integration and decreased global efficiency, we believe that these studies have some shortcomings. First, they analyzed the whole brain network, but different brain networks mainly took on different cognitive task (32), which relied on coactivation of executive network (e.g., frontal–parietal control network) and reciprocal suppression of the nonexecutive network (e.g., default mode network, DMN). The whole brain network analysis may have confused the role of different brain networks. Fair and colleagues found reduced spontaneous activity within the DMN in ADHD (33), and a follow-up study found decreased connectivity in DMN and dorsal attention networks, and enhanced connectivity within reward-motivation regions in the resting-state in young adults with ADHD (34). These previous findings suggest the presence of altered functional brain networks associated with attention and cognitive processing in ADHD. However, the topological features of functional brain networks in FPAN have yet to be extensively investigated. The FPAN is a critical module in attention processing (8), and exploring its alteration in ADHD may be helpful for understanding the pathological mechanism of disease. Second, usually, they used a range of thresholds to construct the SW topology, and the difference between ADHD and typically developing controls (TDCs) mainly exists at some threshold, which was not robust and lost many low signals. In addition, they did not compare with the real random networks. We used the connectivity strength between each pair of brain regions as the edge to construct the SW topology and compared the observed network with randomized networks generated from the observed network to normalize. We also construct MST to explore the alteration of brain networks in ADHD. Conventional network measures may give inaccurate differences in connectivity strength, density, and graph size between subjects. MST overcomes these problems and provides an elegant solution, which, up to this point, has not received much attention in the neuroscience literature. MST is an unbiased approach, and the diameter and leaf number of MST were strongly related with the path length of SW topology (24). MST captured changes in FPAN topology, supporting results derived from conventional network analysis (24). In addition, MST successfully captured alterations in the properties of the whole-brain network during maturation in children (23) and supported the finding that the randomness of the topology reduced with age, as shown by conventional network analysis (24). Finally, although many studies have reported group differences between children with ADHD and TDC, they have not associated these differences with clinical features. In the present study, we computed the Pearson’s correlation between the properties of SW topology and clinical features.

In the present study, we used concepts from graph theory to examine resting-state functional connectivity within the FPAN. We hypothesized that the functional networks in the FPAN would shift towards being more random in children with ADHD than in TDC. We used graph theory analysis to quantify publically available resting functional magnetic resonance imaging (MRI) data from 119 children with ADHD and 69 TDC. We calculated several measures derived from the SW and MST to assess local integration, global efficiency, and relative node importance within the networks and hypothesized that brain networks of participants with ADHD would display lower global efficiency and local integration than brain networks of TDC, and that this would be accompanied by a loss of centrality of individual brain regions within the FPAN. An analysis was then conducted to determine correlations between SW and MST parameters and ADHD-related disability, as measured using the ADHD symptom score. We hypothesized that, in children with ADHD, SW and MST would be associated with ADHD symptom score.

## Materials and Methods

### Participants and Data Acquisition

The data we used in this study are publicly available from the ADHD-200 Consortium (http://fcon_1000.projects.nitrc.org/indi/adhd200/). We first selected 191 participants between the ages of 7 and 14 years from New York University Child Study Center and excluded 7 participants whose IQ (Wechsler Abbreviated Scale of Intelligence, WASI), gender, or diagnosis information were missing, resulting in the 188 participants for further analysis, including 119 children with ADHD (male n = 89; female n = 30) and 69 TDC children (male n = 33; female n = 36), detailed in **Table 1**. All participants provided signed informed consent as approved by the IRBs of NYU and the NYU School of Medicine and were compensated, and the institutional review boards approved the research protocols.

**Table 1:** Demographic and clinical characteristics of ADHD and TDC groups.

	TDC (n = 69)		ADHD (n = 119)		ADHD vs. TDC
	Mean	SD	Mean	SD	t values
Age (years)	10.252	1.935	10.192	1.799	0.217
Handedness	0.568	0.287	0.645	0.291	−1.745
Gender	0.478	0.503	0.748	0.436	−3.715^***^
VIQ	112.594	14.199	107.076	13.890	2.604^**^
PIQ	107.522	15.560	103.941	14.840	1.566
ADHD Index	45.522	5.229	72.261	8.909	−26.739^***^

Handedness, Edinburgh Handedness Inventory; VIQ, Verbal IQ; PIQ, Performance IQ; ADHD Index, ADHD Index Scale T-score. ADHD, attention-deficit/hyperactivity disorder; TDC, typically developing control. *P < 0.05, **P < 0.01, ***P < 0.001.

### ADHD Symptoms Measures

Dimensional ratings of ADHD symptoms (Inattention; Hyperactivity/Impulsivity) were assessed using Conners’ Parent Rating Scale-Revised, Long Version (CPRS-LV).

### Magnetic Resonance Imaging Dataset and Processing

#### Magnetic Resonance Imaging Dataset

High-resolution T1-weighted 3D MPRAGE images covering the whole brain were acquired for each participant on a Siemens 3.0-Tesla Allegra MRI scanner at the NYU Center for Brain Imaging [time repetition (TR) = 2,530 ms, echo time (TE) = 3.25 ms, T1 = 1,100 ms; flip angle = 7°, voxel size = 1.3 × 1.0 × 1.3 mm, field of vision (FOV) = 256 mm]. Functional imaging was performed in a single run using a blood oxygenation level-dependent (BOLD) contrast sensitive gradient echo-planar sequence (TR = 2,000 ms, TE = 15 ms, flip angle = 90°, FOV = 240 mm, 33 slices per volume, 176 volumes, acquisition voxel size = 3.0 × 3.0 × 4.0 mm). During this scan, participants were asked to relax with their eyes open.

#### Data Processing

Image preprocessing was performed using the DPARSF data processing assistant for resting functional MRI (rsfMRI) (35). Preprocessing comprised the following steps: 1) discarding the first 10 volumes; 2) slice timing to correct for temporal offsets; 3) realignment of each volume for head movement; 4) spatial normalization to MNI space (New Segment + DARTEL) and then resampled to 3-mm isotropic voxels; 5) spatial smoothing with a 4-mm 3D full width at half-maximum kernel; 6) detrending to remove linear trends due to scanner drift; 7) temporal band-pass filtering (0.01–0.1 Hz) to remove low-frequency drift and high-frequency physiological noises; and 8) regressing whole brain and white matter signals out of the 24 motion parameters.

### Graph and Functional Connectivity Analysis

**Figure 1:**
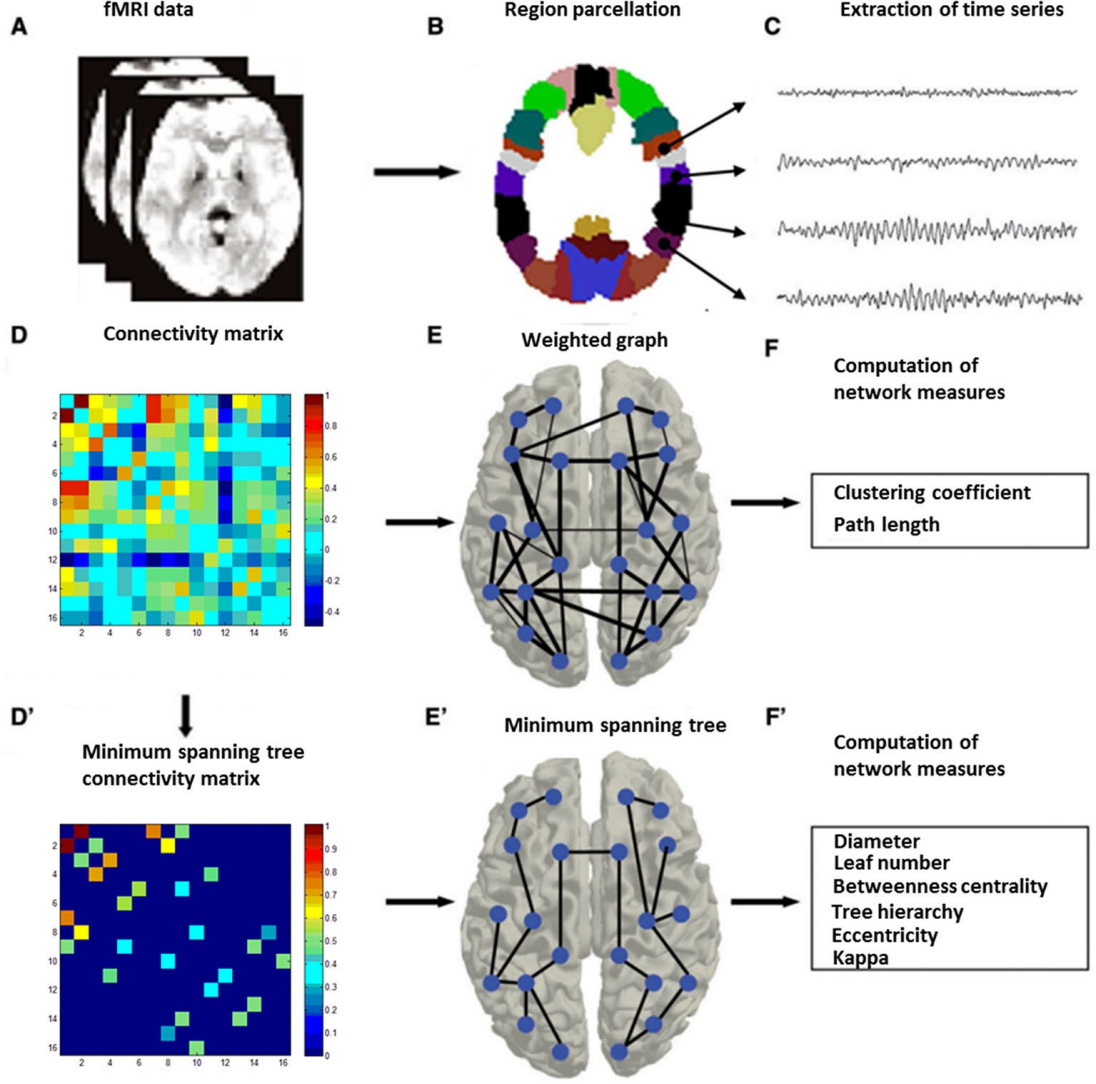
Graph and functional connectivity analysis pipeline. Schematic overview of the formation of individual functional brain networks using two different methods, i.e., construction of weighted graphs and minimum spanning trees (MST). After MRI recording **(A)**, data from the MRI **(B)** were projected onto a functional framework of 16 frontal–parietal attention network (FPAN regions) **(C)**. Functional connectivity between each pair of brain regions was assessed by means of the Pearson’s correlation (Fisher’s r-to-z transformation) **(D)**. For weighted network analysis, a weighted graph **(E)** was constructed from the Pearson’s correlation (z-standardized). For minimum spanning tree analysis, the minimum spanning tree matrix also was derived from the correlation (z-standardized) matrix by Kruskal’s algorithm **(D’)**, with concurrent MST construction **(E’)**. Finally, network measures were computed for both the weighted graph **(F)** and the minimum spanning tree **(F’)** (28).

Graph analysis was performed using Gretna software (36) for BOLD time series extraction (https://www.nitrc.org/projects/gretna) and Brain connectivity toolbox (37) for SW and MST topology (https://www.nitrc.org/projects/bct/). The functional connectivity derived from 16 brain regions forming FPAN (detailed in **Table 2**), which come from previous literature and were transformed to The Montreal Neurological Institute (MNI) coordinates (7, 9). Regions of interesting (ROIs) were defined as 6-mm-radius spheres around these MNI coordinates (8). We extracted BOLD time series from each of the voxels in each ROI, and averaged all voxels in the respective ROI as the signal. The functional connectivity between each pair of ROIs was then computed by a Pearson’s correlation and formed a 16×16 matrix, which were z-standardized by Fisher’s r-to-z transformation to approximate a Gaussian distribution. Typical graph analyses of weighted networks ignored negative correlation (1), and we followed the traditional approach. We used the matrix to construct SW networks and to compute the network properties. Graph and functional connectivity analysis pipelines are shown in **Figure 1**.

**Table 2:** MNI coordinates of the 16 nodes in the FPAN.

Brain region	x	y	z
Left IPS	−23	−70	46
Right IPS	25	−62	53
Left iPL	−42	−48	51
Right iPL	48	−41	54
Left vIPS	−26	−84	24
Right vIPS	35	−85	27
Left FEF	−24	−15	66
Right FEF	28	−10	58
IPCL	−55	−2	38
SMA	−2	−2	55
Left DLPFC	−40	39	30
Right DLPFC	38	41	26
Left vOC	−47	−71	−8
Right vOC	55	−64	−13
Left aIns	−45	35	9
Right aIns	45	3	15

IPS, intraparietal sulcus; IPL, inferior parietal lobule; FEF, frontal eyefield; iPCS, inferior precentral sulcus; SMA, supplementary motor area; DLPFC, dorsolateral prefrontal cortex; vOC, ventral occipital lobe; alns, anterior insula.

### Small-World Properties

In order to approximate a Gaussian distribution, the Pearson’s correlation coefficients in the resulting 16×16 matrix were transformed by Fisher’s r-to-z. This matrix represents the strength of the functional connectivity between all 16 regions in the whole brain and served as an adjacency matrix for graph analysis. SW parameters clustering coefficient (C) and path length (L) were calculated in terms of Watts and Strogat (1998). Briefly, characteristic path length is defined as the average shortest path connecting any two nodes in the graph. The path length is used to measure how well a network is connected; a small value indicates an average short distance between any two nodes. The cluster coefficient is defined as the number of actual edges connecting the neighbors of a node divided by the maximum number of edges possible between neighboring nodes. The cluster coefficient of a network is used to measure how many local clusters exist in the network. A high cluster coefficient indicates that the neighbors of a node are often also directly connected to each other, that is, they form a cluster.

To determine whether a network has SW properties, the values of L and C must be normalized by generated random networks (12). SW networks are characterized by path lengths that are similar to those of comparable random networks (L_random_) but with increased cluster coefficients (C_random_):λ = L/L_random_≈1 and γ = C/C_random_ > 1 (38). Random clustering coefficient and path length derived from the mean of those values from 100 random networks. Each random network was generated by randomly reshuffling the edge weights in the original network (39), which ensures that the node degree and node distribution of the random network are similar to those of the original network.

### Minimum Spanning Tree Reconstruction

The MST of an undirected weighted network is a unique acyclic subgraph that connects all the nodes with the minimum possible link weight. In our analysis, we used the maximum connection strength (correlation matrix) as the edge to construct an acyclic subnetwork, equivalent to a MST as obtained by using the Kruskal algorithm (40). Briefly, all connections are arranged in descending order, then starting from the strongest strength edge, consecutive high strength links were added until all nodes (n) were connected and formed an acyclic subnetwork consisted with n-1 edges (**Figure 1**). If adding a link resulted in the formation of a cycle, this link was skipped.

In terms of the information about the topological properties of the MST, we can compute several measures to characterize the topology of the tree, including the diameter, normalized leaf fraction, kappa (degree of divergence), betweenness centrality, and hierarchy. The diameter, which is the largest distance between any two nodes, is defined as the longest shortest path in the network. The normalized leaf number is defined as the number of nodes with a degree of 1, divided by the maximum number of leaves possible given the size of the tree, and is used to measure the integration in the network (41). A decreased value of the normalized leaf number indicates a decreased global efficiency. Previous studies have found leaf number to be an important network parameter during development, and it is sensitive to the changes in aging (25), autism (42), and Parkinson’s disease (28). Kappa, also called degree of divergence, is used to measure the broadness of the degree distribution. A decreased value of kappa indicates a decreased number of highly connected nodes or “hubs.” Betweenness centrality (BC) of a node is defined as the number of shortest paths between any two nodes passing it, divided by the total number of shortest paths in the network. If BC = 0, the node is a leaf node; if BC = 1, the node is a central node in a star-like network. The BC of a node ranges between 0 and 1. Usually, we used BC_max_, which is the BC of the node with the highest BC in the tree to measure the BC of the tree. A decreased value of BC_max_ in the tree indicates a decreased global efficiency and a decreased “hub” strength. Hierarchy is an indicator of the balance between efficient communication paths and overload of hub nodes, which is defined as

$$T_{H}=\frac{L}{2mBC_{max}}$$

where L is the leaf number, m is the number of vertices −1, and BCmax is the maximum value of betweenness centrality. The value of hierarchy ranges between 0 and 1. If leaf number = 2, tree is a line-like topology, and hierarchy approaches 0. If leaf number = m, tree is a star-like topology, and tree hierarchy approaches 0.5. When the number is between 2 and m, tree hierarchy can have higher values (28).

### Statistical Analysis

Statistical differences in age, handedness, gender, verbal, and performance IQ were evaluated using T test (**Table 1**). Due to the difference only in verbal IQ and gender between TDC and ADHD children, all analyses were also conducted with verbal IQ and gender as covariates. Group differences in graph theory analysis and functional connectivity were examined using analysis of covariance (ANCOVA) in which the main effect of diagnosis was tested with verbal IQ and gender as covariates. Moreover, a partial correlation coefficient was used to assess the relation between network topology (in terms of SW and MST parameters) and ADHD symptom score. Statistical analysis was performed using SPSS 21 (IBM, Armonk, NY). Multiple comparisons were controlled using the false discovery rate (q < 0.05) (43).

## Results

### Group Characteristics

**Table 1** summarizes the characteristics of ADHD and TDC children. No significant group differences were observed in age (*t* = 0.217, p = 0.828), handedness (*t* = −1.745, *p* = 0.082), and performance IQ (*t* = 1.566, p = 0.119); gender (*t* = −3.715, *p* < 0.001) and verbal (*t* = 2.604, *p* = 0.010) IQ showed differences between ADHD and TDC children. In the subsequent analysis, gender and verbal IQ were used as covariates.

### Functional Connectivity

After controlling for gender and verbal IQ, no significant group differences were observed for the FPAN mean strength [ADHD: 0.155 ± 0.034; TDC: 0.153 ± 0.035, *F*(1,184) = 0.472, *p* = 0.493], detailed in **Table 3**. No significant correlation (*r* = 0.023; *p* = 0.754) was observed between the FPAN mean strength and the ADHD scores, detailed in **Table 4**.

**Table 3:** Group differences in network parameters.

		Group*(N)*		Mean ± SD	F-value
Strength		TDC	69	0.154 ± 0.034	0.472
		ADHD	119	0.154 ± 0.033	
SW	*C*	TDC	69	1.221 ± 0.219	.328
		ADHD	119	1.254 ± 0.193	
	*L*	TDC	69	1.104 ± 0.072	1.209
		ADHD	119	1.118 ± 0.066	
MST	*Dia*	TDC	69	0.632 ± 0.099	7.387*
		ADHD	119	0.667 ± 0.089	
	*Leaf*	TDC	69	0.428 ± 0.084	6.098*
		ADHD	119	0.410 ± 0.071	
	*BC*	TDC	69	0.656 ± 0.060	0.724
		ADHD	119	0.649 ± 0.058	
	*Th*	TDC	69	0.022 ± 0.004	5.505*
		ADHD	119	0.021 ± 0.004	
	*Ec*	TDC	69	7.224 ± 1.113	2.959
		ADHD	119	7.410 ± 1.016	
	*K*	TDC	69	2.308 ± 0.157	7.780*
		ADHD	119	2.264 ± 0.123	

Strength, functional connectivity strength; SW, small world; MST, minimum spanning tree; C, clustering coefficient; L, path length; Dia, diameter; BC, betweenness centrality; Th, tree hierarchy; Ec, eccentricity; K, kappa (degree divergence). *P < 0.05, false discovery rate (FDR) corrected.

**Table 4:** Correlations between network parameters and disability score.

	FC	SW		MST					
		*C*	*L*	*Dia*	*Leaf*	*BC*	*Th*	*Ec*	*K*
ADHD Index	0.023	0.040	0.097	0.175*	−0.208*	−0.016	−0.218*	0.112	−0.212*

Strength, functional connectivity strength; SW, small world; MST, minimum spanning tree; C, clustering coefficient; L, path length; Dia, diameter; BC, betweenness centrality; Th, tree hierarchy; Ec, eccentricity; K, kappa (degree divergence). *P < 0.05, FDR corrected.

### Small-World Topology

No significant group differences were observed in SW topology clustering coefficient [ADHD: 1.254 ± 0.193; TDC: 1.221 ± 0.219, *F*(1,184) = 0.328, *p* = 0.568] or path length [ADHD: 1.118 ± 0.066; TDC: 1.104 ± 0.072, *F*(1,184) = 1.209, *p* = 0.273], detailed in **Table 3**. No significant correlation was observed between SW topology clustering coefficient and ADHD symptom score (*r* = 0.040, *p* = 0.590) or path length and ADHD symptom score (*r* = 0.097, *p* = 0.186), detailed in **Table 4**.

### Minimum Spanning Tree Topology

A significant group difference was observed for diameter [ADHD: 0.667 ± 0.089; TDC: 0.632 ± 0.099, *F*(1,184) = 7.387, *p* = 0.007], leaf number [ADHD: 0.410 ± 0.071; TDC: 0.428 ± 0.084, *F*(1,184) = 6.098, *p* = 0.014], tree hierarchy [ADHD: 0.021 ± 0.004; TDC: 0.022 ± 0.004, *F*(1,184) = 5.505, *p* = 0.020], and kappa [ADHD: 2.264 ± 0.123; TDC: 2.308 ± 0.157, *F*(1,184) = 7.780, *p* = 0.006], detailed in **Table 3**. These variables were significantly related to ADHD symptom score (diameter: *r* = 0.175, *p* = 0.017; leaf number: *r* = −0.208, *p* = 0.004; tree hierarchy: *r* = −0.218, *p* = 0.003; kappa: *r* = −0.212, *p* = 0.004), detailed in **Table 4**. No significant group difference was observed for betweenness centrality [ADHD: 0.649 ± 0.058; TDC: 0.656 ± 0.060, *F*(1,184) = 0.724, *p* = 0.396] or eccentricity [ADHD: 0.535 ± 0.028; TDC: 2.308 ± 0.157, *F*(1,184) = 2.071, *p* = 0.152], detailed in **Table 3**, and these variables had no significant correlation with ADHD symptom score (betweenness centrality: *r* = −0.016; *p* = 0.829; eccentricity: *r* = 0.112; *p* = 0.129), detailed in **Table 4**.

To further examine the regionally nodal characteristics of brain networks, the group difference in nodal eccentricity was tested in the MST topology. Eccentricity was significantly greater in children with ADHD than in TDC in the left intraparietal sulcus [ADHD: 7.328 ± 1.595; TDC: 6.783 ± 1.617, *F*(1,184) = 7.017, *p* = 0.009], bilateral ventral intraparietal [left—ADHD: 8.059 ± 1.457; TDC: 7.406 ± 1.584, *F*(1,184) = 11.305, *p* = 0.001; right—ADHD: 7.79 ± 1.545; TDC: 7.304 ± 1.365, *F*(1,184) = 9.206, *p* = 0.003], and left and right ventral occipital lobe [left—ADHD: 7.731 ± 1.655; TDC: 7.087 ± 1.755, *F*(1,184) = 6.143, *p* = 0.014; right: ADHD: 7.462 ± 1.736; TDC: 6.768 ± 1.637, *F*(1,184) = 8.809, *p* = 0.003], which also correlated with ADHD scores (left IPS: *r* = 0.165, *p* = 0.025; left vIPS: *r* = 0.205, *p* = 0.005; right vIPS: *r* = 0.154, *p* = 0.036; left vOC: *r* = 0.195, *p* = 0.008; right vOC: *r* = 0.172, *p* = 0.019), detailed in **Table 5**.

**Table 5:** Regions showing significant changes in each nodal eccentricity in ADHD.

	Group*(N)*		Mean ± SD	F-value
Left IPS	TDC	69	6.783 ± 1.617	7.017*
	ADHD	119	7.328 ± 1.595	
Left vIPS	TDC	69	7.406 ± 1.584	11.305*
	ADHD	119	8.059 ± 1.457	
Right vIPS	TDC	69	7.304 ± 1.365	9.206*
	ADHD	119	7.79 ± 1.545	
Left vOC	TDC	69	7.087 ± 1.755	6.143*
	ADHD	119	7.731 ± 1.655	
Right vOC	TDC	69	6.768 ± 1.637	8.809*
	ADHD	119	7.462 ± 1.736	

IPS, intraparietal sulcus; vOC, ventral occipital lobe. *P < 0.05, FDR corrected.

## Discussion

To our knowledge, this is the first study to investigate SW and MST properties of FPAN topology in children with and without ADHD. We found that, although brain functional networks exhibited economical SW topology in both groups, children with ADHD had greater MST diameter and lower leaf number, tree hierarchy, and kappa than TDC, and these variables were also associated with ADHD symptom score.

Since Watts and Strogatz proposed and quantitatively described SW networks (12), it has been applied in brain structural and functional networks in many studies using various imaging techniques including electroencephalography, magnetoencephalography, and functional MRI (13, 44). Wang and colleagues first explored SW topology in the whole-brain functional network in ADHD and found SW topology in TDC and children with ADHD, but children with ADHD had a tendency towards more regular networks. Consistent with previous studies (21, 45), we found that the FPAN had economical SW properties, which suggests that SW brain networks are robust in the face of disease (21). This supports the view that brain networks may have developed to maximize the efficiency of information processing. However, we did not find significant alterations in FPAN in children with ADHD. It may be that children with ADHD had no deficit in FPAN topology, or that any difference was too small to be captured by clustering coefficient and path length. Previous studies have used one of two approaches for normalizing clustering coefficient and path length. However, thresholding the functional connectivity matrix cannot provide a unique or consistent solution (27, 46). We used the second approach to normalize, whereby the observed network parameters were divided by the randomized networks parameters, but this approach may include too many low noise and may not be sensitive to developmental disease. MST is an unbiased approach that overcomes the normalization problem, can provide a unique and consistent solution, and can discard the low signal.

In the present study, MST analysis showed that diameter, leaf number, tree hierarchy, and kappa were altered in ADHD. Children with ADHD had greater diameter and lower leaf number, tree hierarchy, and kappa, and these variables significantly correlated with ADHD symptom score, indicating their clinical relevance. Decreased leaf number and increased diameter indicate a decreased global efficiency (47), suggesting that the FPAN had lower global efficiency in children with ADHD than in TDC. This is consistent with whole-brain deficits (21). Together with the significant negative correlation between ADHD symptom score and leaf number, and positive correlation between ADHD symptom score and diameter, this indicates the tendency to deviate from a more centralized organization (star-like topology) towards a more decentralized organization (line-like topology) in ADHD. The negative correlation between ADHD symptom score and tree hierarchy suggests that there is a sub-optimal balance between hub overload and functional integration in the network. Tree hierarchy can range from 0 to 1, and an optimal tree configuration is thought to correspond to a hierarchy value of around 0.5 (a compromise between a line-like and star-like topology). A star-like topology corresponds to hub overload, and a line-like topology corresponds to weak integration (32). The lower tree hierarchy in children with ADHD represents a more line-like topology, which is indicative of weak integration. This is consistent with the finding of a study based on whole-brain analysis that reported a decreased clustering coefficient, which corresponds to a local integration, in children with ADHD (48), in which they found a decreased clustering coefficient (corresponds to a local integration). Using MST analysis, we confirmed the decreased integration in children with ADHD. In addition, kappa, a measure that captures the broadness of the degree distribution, was lower in children with ADHD than in TDC and was negatively associated with ADHD symptom score. The lower kappa in children with ADHD reflects a reduced ease of synchronization, that is, decreased spread of information across the tree (41). As hypothesized, these findings indicate that FPAN topology is different in children with ADHD and TDC, and tends towards greater randomness and lower global efficiency and local integration in children with ADHD. The correlation between MST parameters and ADHD symptom score suggests that the abnormal MST topology may be useful in monitoring progression of the disease.

A low kappa value corresponds to a low number of highly connected nodes or “hubs.” The number of hubs is associated with the resilience of the network against attack. To further explore the damaged “hub” regions in the FPAN in children with ADHD, we further computed the eccentricity of each region. The eccentricity of a node is measured by the longest distance between that node and any other node. The closer a node is to the center of the tree, the lower its eccentricity. Low eccentricity indicates high global efficiency and centrality. We found greater eccentricity, reflecting lower global efficiency and centrality, in the left IPS, bilateral vIPS, and bilateral vOC. In general, these brain regions were concerned in ADHD studies. Previous studies found that the centrality in the IPS and vIPS regions of the FPAN was associated with alertness and the efficiency of the executive control system (8, 49). Consistent with the results of these studies, the decreased centrality in the IPS and vIPS in children with ADHD may be related to altered alerting and executive function in attention processing. These findings are in accordance with those of several structural and functional imaging studies that have found cortical atrophy and reduced activity in these regions in participants with ADHD (49). In addition, vOC also shows lower eccentricity in ADHD participants, which were compatible with previous studies that found decreased nodal efficiency and reduced volume in this region (21, 50).

In summary, this is the first study to reveal the topological properties of the FPAN in children with ADHD using resting-state functional MRI. We performed MST analysis of brain networks. This addresses the threshold and normalization problems encountered with conventional approaches, and was sensitive to changes in brain topography in children with ADHD. Clustering coefficient and path length were not successful in identifying deficits in the FPAN, whereas the MST parameters of leaf number, diameter, tree hierarchy, and kappa captured the tendency of ADHD brains to deviate from a more centralized organization (star-like topology) towards a more decentralized organization (line-like topology). This corresponds to a decreased global efficiency and weak integration. There were also differences in nodal eccentricity of the IPS, vIPS, and vOC in children with ADHD, reflecting a decreased efficiency and decentralized topology that was associated with deficits in alertness and executive function in attention processing. In addition, MST parameters were associated with clinical features of ADHD. These findings enhance our understanding of the underlying pathophysiology of ADHD and may facilitate evaluation and monitoring of clinical status in ADHD.

Despite the advantages of this study, some limitations should be noted. First, when using MST, we only used the “core” connections. This means that some information may have been lost. For example, clustering coefficient is a measure that cannot be examined in MST. To address this, we derived this information from SW topology. Previous studies found that, among children with ADHD, there are gender differences in brain structure (51); however, girls comprised only 35% of our participants. In addition, participants in the ADHD and TDC groups were not matched for gender or IQ. To address this limitation, we treated these variables as covariates; however, future studies should further explore differences in brain networks using participants matched for IQ and gender.

## Ethics Statement

This study was carried out in accordance with the recommendations of the IRBs of NYU and the NYU School of Medicine with written informed consent from all subjects. All subjects gave written informed consent in accordance with the Declaration of Helsinki. The protocol was approved by the IRBs of NYU and the NYU School of Medicine.

## Author Contributions

YW analyzed the data and wrote the draft of the paper. CZ and FT amended and proofread the draft of the paper. CZ, FT, DW, MH, and MK participated in the discussion and offered some good ideas. All authors reviewed the manuscript.

## Funding

This research was supported by the National Natural Science Foundation of China (No. 31662083). 

## Conflict of Interest Statement

The authors declare that the research was conducted in the absence of any commercial or financial relationships that could be construed as a potential conflict of interest.
